# How much can healthier diets reduce future economic and human costs? Results from Ethiopia and the Philippines

**DOI:** 10.1093/heapol/czag018

**Published:** 2026-02-10

**Authors:** Susan Horton, Michelle F Gaffey, Felipe Dizon, Eldridge Ferrer, Maria Julia Golloso-Gubat, Giles Hanley-Cook, Kristine Nacionales, Kyoko Shibata Okamura, Patrizia Fracassi

**Affiliations:** School of Public Health Sciences, University of Waterloo, 200 University Avenue West, Waterloo, Ontario, Canada N2L 3G1; Food and Agriculture Organization of the United Nations, Via delle Terme di Caracalla, 00153 Rome, Italy; World Bank Group, 1818 H Street NW, Washington, DC 20433, USA; Department of Science and Technology, Food and Nutrition Research Institute, DOST Compound, General Santos Avenue, Bicutan, Taguig City, Philippines; Department of Science and Technology, Food and Nutrition Research Institute, DOST Compound, General Santos Avenue, Bicutan, Taguig City, Philippines; Food and Agriculture Organization of the United Nations, Via delle Terme di Caracalla, 00153 Rome, Italy; Department of Science and Technology, Food and Nutrition Research Institute, DOST Compound, General Santos Avenue, Bicutan, Taguig City, Philippines; World Bank Group, 1818 H Street NW, Washington, DC 20433, USA; Food and Agriculture Organization of the United Nations, Via delle Terme di Caracalla, 00153 Rome, Italy

**Keywords:** unhealthy diets, noncommunicable disease, overweight and obesity, high systolic blood pressure, high fasting blood glucose

## Abstract

As countries progress through the ‘nutrition transition’ and experience rising rates of obesity and noncommunicable disease, concern has broadened from a primary focus on economic consequences of child stunting, to incorporate multiple forms of malnutrition, including overweight and obesity, or a more ambitious set of individual dietary risk factors from the Global Burden of Disease work. This paper conceptualizes a methodology that uses unhealthy diets to better understand the economic impact as the nutrition transition progresses. The Lives Saved Tool (LiST) is used to estimate how much healthier diets alone (without other health interventions) can reduce future child stunting. The Global Burden of Disease Results Tool is used to estimate how much healthier diets can reduce future noncommunicable disease among adults, via effects on three metabolic markers (high body mass index -BMI, high systolic blood pressure, and high fasting blood glucose). We then link the metabolic markers to diet quality (measured by the Global Diet Quality Score). Calculations are made for the Philippines for 2014 and 2021 and Ethiopia for 2011 and 2019. Recent studies have estimated the present value of future child stunting costs as 2.0% of GDP for the Philippines and 5.25% for Ethiopia, in both cases in 2023, of which we estimate up to 45% and 50%, respectively, are avertible over the long run by healthier diets, while public nutrition and public health programs account for the rest. The present value of costs associated with the three metabolic markers among adults is estimated as 7.99% of GDP (Philippines 2021) and 2.15% (Ethiopia 2019), of which we estimate 20% is avertible by healthier diets. The total losses avertible by healthier diets are therefore estimated as 2.5% of GDP (Philippines 2021) and 3.1% (Ethiopia 2019), with metabolic factors predominating in the Philippines and stunting in Ethiopia.

Key messages

- Unhealthy diets impose important societal costs, both when nutrients are inadequate and children fail to reach their full growth potential, and when dietary patterns increase the risk of noncommunicable disease
- Existing studies do not accurately capture these costs: child stunting does not only depend on diet; healthy diet and healthy weight in adults are not synonymous; and adding up known effects of individual dietary risk factors involves an unknown amount of double-counting
- Further research using holistic measures of a healthy diet will be useful, as will incorporating additional metabolic markers such as blood pressure and blood glucose into national nutrition surveys
- The focus on the healthiness of diets rather than nutritional status highlights the importance of agrifood policies to complement public health policies

## Introduction

Unhealthy diets are a greater health risk than unsafe sex, alcohol, and tobacco combined (Willett et al. 2019). The estimates of the losses to human health and to economic outcomes are very large, although they vary according to both the definition adopted of unhealthy diets, and the methodology used. The FAO-WHO Joint Statement (2024) lists four key principles of healthy diets, namely that they should be adequate, balanced, moderate, and diverse. Studies using nutritional status as a proxy for unhealthy diets have estimated global costs of stunting as 1% of global income (Nutrition International 2025), and of obesity and overweight as 2.2% in 2019 (Okunogbe et al. 2022, for 160 countries), with mortality costs of 1.3 m deaths (Nutrition International 2025) and 4.7 m deaths in 2017 (Dai et al. 2020), respectively. Other studies using individual diet risk factors estimate the global costs as 9.2% of global GDP in 2020 (FAO 2024) and 11 m deaths in 2017 (GBD 2017 Diet Collaborators 2019), i.e. considerably higher.

The present study examines the societal cost of unhealthy diets in two countries at two points in time. As with previous costing studies (e.g. Fernández et al. 2008, African Union and WFP 2020), we are projecting forward what costs would be if diets were healthier, compared to a continuation of the status quo. A variant on previous methodologies is used, since nutritional status is not an ideal proxy for the healthiness of diet, and since using a more holistic measure of diet quality avoids the potential double-counting inherent in adding up risks of individual diet components. We first take existing estimates of costs of child stunting and then use the LiST model (Avenir Health 2025) to project forward two different policy paths in order to parse out the component attributable to diet (as opposed to infection), for those diets where the paramount limiting factor is inadequacy of nutrient intake. We then utilize the GBD Results Tool (IHME VizHub 2021) to estimate the risks associated with three key metabolic markers among adults (high body mass index—BMI, high systolic blood pressure, and high fasting blood glucose) in order to estimate health impacts and hence costs, and then in turn attempt see how country Global Diet Quality Scores (Intake 2022) affect these markers. Our results are then compared to those using two different previously published estimates (Okunogbe et al. 2021, FAO 2024) for the same two countries. The methodology employed was initially developed in World Bank and FAO (2025) and applied to data from more than a decade ago. This paper extends that work to more recent data to examine the evolution of costs over approximately a decade in each country as the nutrition transition proceeds, and also by making the link to a holistic, empirical measure of diet quality in the Philippines.

The empirical literature to date has tended to focus on upper-middle and high-income countries, which have greater availability of food consumption and nutrition surveys. The interest here is to examine these issues in a lower-income context: it has proven difficult to reverse obesity and noncommunicable disease trends once established, and there is therefore some urgency to addressing policy issues earlier on. Country-level data can be useful for bringing the issue to the attention of national policymakers. The two country examples used are Ethiopia and the Philippines, each for two points in time. These countries have advantages of available food and nutrition surveys. The surveys date back to 1978 in the Philippines (Patalen et al. 2020), while Ethiopia has a national food consumption survey from 2011 (Ethiopian Public Health Institute 2013) and a national food and nutrition survey with a baseline report published in 2023 (Ethiopian Public Health Institute 2023). The countries provide contrasts in that the Philippines is currently in the process of transitioning from lower-middle to upper-middle income status, while Ethiopia is transitioning from low to lower-middle income status. The two countries are also transitioning from a traditional diet high in fibre, to the more Western pattern diet high in sugars, fat, and animal source food, termed by Popkin (1993) the nutrition transition. Ethiopia could be regarded as being in stage three of Popkin’s five stages, the ‘receding famine’ stage, while the Philippines could be regarded as being in stage four, i.e. ‘degenerative diseases’: the country context section provides more evidence on this transition.

The next section discusses the conceptual model underpinning the methods, describes the steps in the costing, and the context in the two countries.

## Materials and methods

### Conceptual models

**Figure 1:**
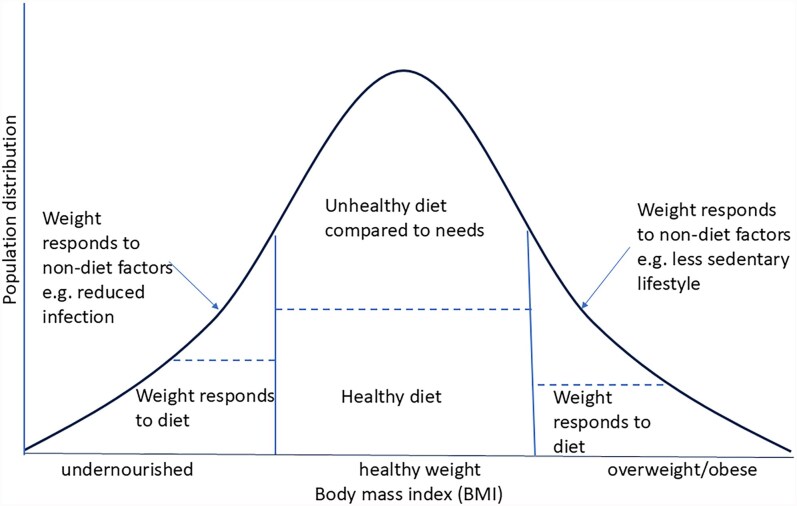
Nutritional status is an imperfect proxy for dietary intake. Source: These authors, for World Bank and FAO (2025).

Figure 1 illustrates why neither stunting nor BMI are perfect metric of dietary risks, and how both are affected by other factors. Weight gain as children grow depends on nutrient intake but can also be adversely affected by infection, which in turn depends on public health measures such as vaccination and water and sanitation. Stunting of children below the age of five is the commonly used measure of long-run nutritional status, and literature on the first 1000 days (pregnancy and the first two years of life: World Food Program 2025) suggests that this is the crucial window in which stunting is established.

Adults with a healthy weight (BMI) do not necessarily have a healthy diet; e.g. they may consume more salt than recommended by nutritional guidelines and, hence, face elevated risks of high blood pressure and cardiovascular conditions. Finally, exercise and stress levels are known to be important for maintaining a healthy weight for adults. While it is critical to establish healthy lifestyle habits between ages 6 and 18, the adverse consequences are not usually visible until later in life.

**Figure 2:**
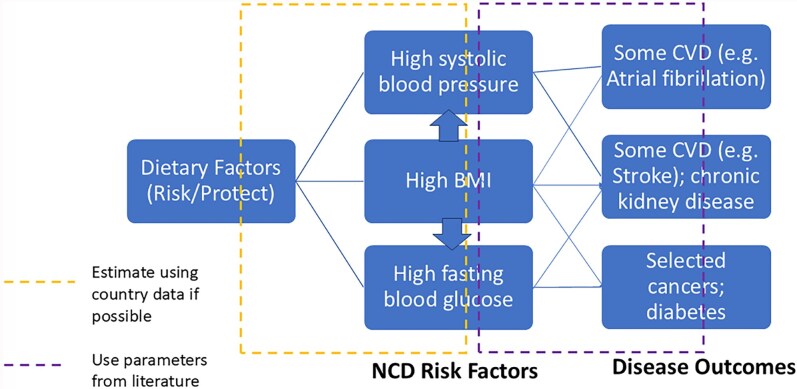
Simplified conceptual model of dietary impact on NCDs. Source: These authors, for World Bank and FAO (2025). CVD denotes cardiovascular disease.

Figure 2 is a simplified conceptual model of how unhealthy diets affect societal costs. Unhealthy diets are associated with higher levels of three metabolic markers (ideally estimated with country data, shown by an orange box in the figure), which in turn are associated with increased NCD risks (which can be estimated using multicountry data, shown by a purple box in the figure). There are also interactions among the metabolic markers, with overweight and obesity being correlated with increased levels of high blood pressure and high blood glucose. According to GBD 2021 Risk Factor Collaborators (2024), high systolic blood pressure affects eight conditions, high fasting blood glucose 10 in addition to 6 cancers (as well as tuberculosis for people living with diabetes), and high BMI affects 16 conditions plus at least 13 cancers plus tuberculosis. Other factors (not included in the figure) also affect metabolic markers (e.g. exercise) and have direct effects on disease risk (e.g. smoking and genetic factors). Healthiness of the diet is assessed here by a single metric (the sum of two submetrics) to avoid the double-counting implicit in aggregating across multiple individual diet components.

This study uses two different models, one to analyse the contribution of unhealthy diets to child stunting, and the other to analyse the contribution of unhealthy diets to adult noncommunicable disease. Economic costs are estimated for both cases, each for two countries at two points in time.

### Obtaining estimates of child stunting attributable to diet

The Lives Saved Tool (LiST: https://www.livessavedtool.org) was used to estimate the proportion of stunting avertible by healthy diets, as opposed to other factors. The LiST model was initially developed at Johns Hopkins University and is now maintained by Avenir Health (2025) as a component of their Spectrum software package. The model is based on evidence reviews and models the effect on child mortality and child stunting of a variety of interventions as coverage levels change. A ‘healthy diet’ is not represented by a single intervention. Rather, the present study uses a set of interventions applied both to women during peri-conception and pregnancy, and to children below the age of five. The four diet interventions (Bhutta et al. 2013) are:

**Diet**

- Fortified balanced energy-protein supplementation in pregnancy;
- Exclusive breastfeeding among children aged 0–5.9 months;
- Appropriate complementary feeding among children aged 6–23.9 months; and
- Food fortification with folic acid and iron reaching women during periconception.

The intervention during pregnancy is defined in LiST as ready-to-use food products that provide multiple micronutrients and, specifically, energy and protein in a balanced composition (<25% of total kcal content from protein) to address maternal malnutrition during pregnancy and lactation. These are recommended in populations where the prevalence of maternal underweight exceeds 20%. While these four interventions are not identical to a healthy diet, they aim to model the major effects of a healthy diet on the current child generation. Bhutta et al. (2013) also include two treatment interventions for severe and moderate malnutrition, which are not included here since the focus is on prevention. Eliminating stunting typically takes several generations, as child weight and length at birth also depend on maternal height.

Again, following Bhutta et al. (2013), four public nutrition interventions affecting stunting were included as follows (combined here into two interventions):

**Public nutrition**

- Iron and calcium supplementation in pregnancy; and
- Vitamin A and zinc supplementation among children aged 6–59 months and 12–59 months, respectively.

Bhutta et al. (2013) focused on the effect of nutrition-specific interventions on child stunting, which did not include broader public health measures. The synthesis of systematic reviews conducted by Bhutta et al. (2013) suggested that WASH, malaria control, and diarrhoea-control interventions (represented in LiST by rotavirus vaccination) have significant effects on stunting. We added three key components of antenatal care, since a recent empirical study suggested use of antenatal care across 52 countries significantly affects birthweight (Teklu et al. 2023), also confirmed by a systematic review for Africa (Engdaw et al. 2023). All these public health measures are available in LiST.

**Public health, including WASH**

- Syphilis detection and treatment, progesterone for high-risk births, and low-dose aspirin in pregnancy;
- Improved drinking water, improved sanitation, and handwashing with soap;
- Rotavirus vaccination; and
- Malaria control (in countries where malaria is endemic).

The modelling examined the impact on stunting by 2030 of increasing levels of coverage of all these interventions from current levels (based on most recent household survey estimates, from 2022 for the Philippines and 2019 for Ethiopia: see Supplementary Table S1) to 100% from 2025 onward. Coverage for six years ensured that all the children in the model cohort in 2025 reaching the age of five in 2030 had received the benefits of all the interventions *in utero* and throughout early childhood. These outcomes were then compared to stunting levels if current levels of coverage were maintained over the same period for the same birth cohort.

Model input data on population dynamics, risk factors, and mortality come from national sources, collated by international organizations such as UNICEF, WHO, and others. Data on coverage of interventions come from sources such as the Demographic and Health Surveys (DHS; www.dhsprogram.com) and Multiple Indicator Cluster Surveys (MICS; https://mics.unicef.org/).

### Obtaining estimates of the human costs of noncommunicable disease

Annual estimates from the GBD 2021 (2024) study of deaths and years of life lost (YLLs) due to each of the three metabolic markers, by cause of death, age group, and country, are available online from the GBD Results Tool (https://vizhub.healthdata.org/gbd-results/). This tool allows users to select and download GBD estimates for one or more measures of the cause-specific morbidity and/or mortality associated with one or more risk factors, for the locations, age groups, sexes, and years of interest. Data on disability-adjusted life years (DALYs) are also available. YLLs were used here as the losses due to premature death are more readily understood than years of life lived with disability when translated to policy discussions, and the choice between YLLs and DALYs does not make a big difference to the findings.

When extracting data and YLLs from the GBD Results tool, the three metabolic markers used here are high BMI, high systolic blood pressure, and high fasting blood glucose for adults 20 and older for each of the years 2011 and 2019 (Ethiopia) and 2014 and 2021 (the Philippines).

### Estimating the economic costs of stunting and noncommunicable disease

The methodology employed here was to estimate the major components of societal costs using USD at market exchange rates, employing methods similar to those of previous studies (e.g. Fernández et al. 2008, African Union and WFP 2020, Okunogbe et al. 2021). No new estimate was made of the costs of stunting, given that more comprehensive studies exist for both Ethiopia (Ethiopian Public Health Research Institute 2013, estimates for 2009) and the Philippines (Save the Children 2016, estimates for 2014), and an online calculator exists which can be used to make estimates for recent years (developed by Jain et al. 2024). All these studies have similar methodology, aiming to estimate the present value of productivity losses, including losses associated with premature mortality and with lower schooling achievement, and hence adult productivity. The only innovation here was to aim to estimate the proportion of stunting costs attributable to diet, and the proportion attributable to public health and public nutrition interventions.

For the economic costs of unhealthy diets in adults, we estimate the present value of mortality attributable to elevated values of the three metabolic factors, which are sensitive to diet, along with treatment costs for those conditions.

To estimate the total economic cost of premature mortality in the base year attributable to each metabolic marker in each country, we multiplied YLLs (obtained from the IHME VizHub 2021) by per capita GDP of the base year (obtained from the World Bank World Data 2025). Use of per capita GDP followed Okunogbe et al. (2022) and gave equal value to all life years irrespective of age and labour market participation status. To calculate the present value of future YLLs, a 3% discount rate per year was applied to account for time preference (as recommended by Bertram et al. 2021) and a per capita GDP growth rate of 3% was assumed, which offset the 3% discount rate. Ethiopia’s GDP per capita was growing at around 3% 2020–2022, and that of the Philippines at just above 3% between 2012 and 2022 (except for a large decline in 2020 associated with COVID-19) (World Bank World Data 2025). Obviously, the present value of costs would be higher in economies growing faster than 3% per capita per annum, and correspondingly lower in slower-growing economies.

According to GBD 2021 Risk Factor Collaborators (2024), metabolic factors (along with nonmetabolic factors) are risks for major categories of NCD, including cardiovascular disease, many cancers, chronic kidney disease, and diabetes, as well as individual conditions such as glaucoma, cataracts, tuberculosis (in individuals living with diabetes), and gallbladder disease. Tuberculosis is included in the calculations for completeness as it is the only communicable disease for which the metabolic markers are a risk factor, included in GBD 2021 Risk Factor Collaborators (2024). There are also NCDs for which diet is not known to be a risk, including respiratory disease, mental and neurological disease (other than Alzheimer’s and dementias), gastric disease (other than gallbladder disease), and nephritis (other than chronic kidney disease). It is assumed that the share of the three metabolic factors in total NCD treatment costs is the same as the share of the three metabolic factors in total NCD deaths, neglecting the very small effect on tuberculosis treatment costs and deaths.

Data on the share of health expenditures in GDP in both countries came from WHO (2024), which also provides data on the share of treatment costs by disease in the Philippines. WHO (2024) does not currently provide data disaggregated by disease condition for Ethiopia, which was instead obtained from Ministry of Health, Ethiopia (2017) and Ministry of Health, Ethiopia (2022).

Okunogbe et al (2021) estimated additional economic costs in their study of overweight and obesity, including out-of-pocket expenditures on treatment, costs of presenteeism (reduced productivity in the workplace due to morbidity), and costs of absenteeism from work. Costs of absenteeism and presenteeism relied on a single study for Brazil, which may not be appropriate for low- and lower-middle-income countries with more limited coverage of sick pay. Okunogbe et al. (2021) estimated travel costs for seeking treatment as the major driver of out-of-pocket costs; however, in countries without national health insurance programmes, this may underestimate out-of-pocket costs. In their analysis, the two major cost components (treatment cost and premature mortality) accounted for 78.5% of total costs. Accordingly, in the present study, total costs were estimated as 1.27 times (1/0.785) the sum of treatment costs and costs of premature mortality to estimate these additional costs.

To make comparisons with other studies, costs are presented as a percentage of GDP, since FAO (2024) uses purchasing power parity (PPP) estimates of dollar costs rather than estimates using market exchange rates. GDP estimates both in PPP and at market exchange rates came from World Bank World Data (2025).

### Attributing economic costs associated with metabolic markers to unhealthy diets

The Global Diet Quality Score (GDQS: Intake 2021, Intake 2022) was used to attribute metabolic risks to diet quality. This metric ranges from 0 to 49 and is the sum of a healthy submetric, the GDQS+, which ranges from 0 to 32, and an unhealthy submetric, the GDQS−, which ranges from 0 to 17. The GDQS has been validated against nutrient intake, nutritional status, and NCDs (Bromage et al. 2021), and its advantages compared to selected other metrics are discussed by an expert evaluation (Verger et al. 2023). Hanley-Cook et al. (2024) suggest that separating the potential effect of the negative submetric of composite dietary metrics provides more actionable information, which in this case would be the GDQS− (ideally mutually adjusted for GDQS+).

A literature survey identified studies for three upper-middle and high income countries linking the GDQS and GDQS- to one or more of the metabolic markers, providing an estimate of the size of the coefficient linking metabolic markers to diets, which (along with information on the distribution of diet quality across the population) can then be used to make an estimate of the proportion of costs of the metabolic risks potentially attributable to diet.

It will be important in future work to examine whether the estimated coefficient also applies in low- and lower-middle-income countries. The Philippines National Nutrition Survey (Patalen et al. 2020) is (to the authors’ knowledge) the only national survey for a lower-middle-income country that collects diet recall data as well as both blood pressure and blood glucose data, and there is no such national survey for a low-income country. Research using this dataset will be important in showing whether the results from upper-middle and high-income countries also apply in other countries. This is taken up in the discussion section.

### Country context

The Philippines faces a double burden of malnutrition. Child stunting decreased from 36.3% in 1998 (UNICEF 2023a) to 26.7% in 2021 (UNICEF 2022), both levels categorized by the WHO as of high public health significance (WHO 2025). The Philippines is one of the ten countries worldwide with the highest number of stunted children. At the same time, levels of overweight and obesity have been increasing. For women 20–49 years old, the increase was from 17.9% in 2000 to 24.6% in 2016 (UNICEF 2023b). Similar trends, albeit at different levels for adolescents, men and older adults can be seen in DOST-FNRI (2022) and using surveys from earlier years cited in Patalen et al. (2020). The true prevalence of hypertension in adults 20 and above rose from 16.4% in 2003 to 20.6% in 2008 but subsequently declined slightly to 19.8% (DOST-FNRI 2022), potentially affected by increased coverage of antihypertensives. Levels of high fasting blood glucose in adults 20 and above, by contrast, more than doubled from 3.9% in 1998 to 8.7% in 2018/19 (DOST-FNRI 2022).

Analysis by FAO et al. (2024) showed that 48.1% of the population in the Philippines in 2022 could not afford a ‘healthy’ diet (defined as conforming to WHO recommendations), unchanged from 2017. This was higher than the regional average for Southeast Asia for the same years (36.3% and 38.4%, respectively). An important factor is linked to limited access and availability of perishable nutritious food due to inefficiencies in the logistics and cold storage systems, in part due to the complexities of being an archipelago (Chong et al. 2021). Only a small proportion of the population consumes a high level of fruit; consumption of high levels of vegetables decreases with urbanization, while consumption of high levels of unhealthy foods such as sugar-sweetened beverages, sweets, and ice-cream increases with urbanization (World Bank and FAO 2025). Rice, a refined grain, is the main staple and provides 54.5% of the energy intake of adults (Galang 2022).

In Ethiopia, the share of under-five children who are stunted fell from 57% in 2000 to 36.8% in 2019 (UNICEF 2023a). Overweight and obesity is still at low levels but increasing, and reached 4.5% of the adult population aged 15–49 in 2016 (3% in men and 8% in women, having risen from 3% in women in 2000: data are not provided on trends in men, and are not available for those aged 6–14 or 50 and over); rates are higher in urban and peri-urban than in rural areas (Central Statistical Agency and ICF 2016). There are no national surveys measuring hypertension and blood glucose, but the share of NCDs in health expenditures increased markedly between 2013/4 and 2019/20 (Ministry of Health, Ethiopia 2022). Starchy staples account for 71.4% of calorie intake nationally (Federal Democratic Republic of Ethiopia 2021). Tef and sorghum (both typically consumed as whole grains) are traditional staples, but production and consumption of rice and wheat are growing (increasingly consumed in refined form in urban areas). Dietary diversity is very low: only 8% of children aged 6–23 months and only 7% of women consumed the minimum number of recommended food groups per day (5 out of a possible total of 8, for both groups), and 3 out of 4 children 6–23 months did not consume any fruit or vegetables per day (Ministry of Health, Ethiopia et al. 2023).

A study by FAO et al. (2023) compared the cost of healthy diets (i.e. with lower risk of NCDs), nutritious diets (meeting minimum nutrient needs), and diets that simply met minimum energy needs. Ethiopia had the third-highest cost of a healthy diet in sub-Saharan Africa in 2021, and 83.9% of the population (over 100 million people) could not afford this diet. Calculations showed that a nutritious diet costs four times as much as a diet which met minimum energy needs, but only one in four families in Ethiopia (modelled as two adults and three children) could afford even the nutritious diet (Federal Democratic Republic of Ethiopia 2021). Postharvest losses of fruits and vegetables are high (up to 45%: Federal Democratic Republic of Ethiopia 2021), a factor in reducing the diversity of diets and causing higher prices.

The dates selected for the calculations using NCDs were 2013 and 2021 for the Philippines, and 2011 and 2019 for Ethiopia, with the aim of illustrating both how costs compare across two countries at different stages of the nutrition transition, and how costs are evolving over recent years. This was based on the availability of national nutrition survey results for 2013/2014 (DOST-FNRI 2015) and 2018, 2019, and 2021 for the Philippines (DOST-FNRI 2022), and 2011 for the national food consumption survey for Ethiopia (Ethiopian Public Health Institute 2013) and 2022 (Ethiopian Public Health Institute 2023). For the Philippines, data on the share of NCD treatment costs in health expenditure is available from 2014 onwards (WHO 2024), and for Ethiopia, for the two financial years 2013/14 (Ministry of Health, Ethiopia 2017) and 2019–20 (Ministry of Health, Ethiopia 2022), but not for prior years. Other constraints were that the GBD 2021 Risk Factors Collaborators (2024) estimates using the metabolic risk factors had been updated at the time of writing only to 2021.

## Results

### Economic costs of stunting attributable to unhealthy diets

**Figure 3:**
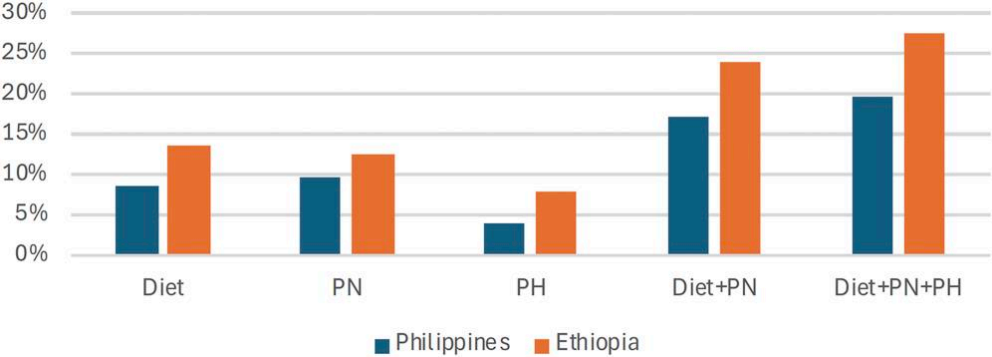
Percentage of stunting that could be averted by diet, public nutrition (PN), and public health (PH) (including WASH), Philippines and Ethiopia. Source: These authors, for FAO (2024).

Table 1 summarizes the economic costs of stunting in the Philippines and in Ethiopia, for 2023 and a previous year, a decade or so earlier for each country (as close as possible to the dates used for the calculations for NCDs), based on previous studies. These costs fell from 2.83% of GDP in the Philippines in 2013 to 1.9% in 2023, and from 15.54% in Ethiopia in 2009 to 5.9% in 2023. Figure 3 provides estimates using the LiST model as to how far stunting is avertible by diet, using simulations of increasing coverage of the various interventions from 2023 levels to 100%, for one birth cohort.

**Table 1:** Costs of stunting, Philippines and Ethiopia, by years and authors.

Country, year	Cost, $bn USD	Cost, % GDP or GNI	Source, details
Philippines, 2013	$18.2 (PPP)	2.83%	Save the Children (2016); stunting was 30.0% in 2013 (UNICEF 2023a, modelled estimates)
Philippines, 2023	$8.1	1.9%	Nutrition International 2025; Stunting was 26.7% in 2021 (UNICEF 2022)
Ethiopia, 2009	$4.7	15.54%	African Union and WFP, 2020; stunting was 44.2% in 2011 (African Union and WFP, 2020),
Ethiopia, 2023	$7.5	5.9%	Nutrition International 2025; stunting was 36.8% in 2019 (UNICEF 2023a)

***Note*:** Costs in $bn are costs in dollars using market exchange rates, except for the Philippines in 2013, where purchasing power parity (PPP) exchange rates are used, of the year of the calculation: cost as % GDP or GDI is a better basis for comparison. Stunting data are for children under five.

The estimates suggest that diet alone can avert 9% of stunting in one generation in the Philippines, compared to 14% in Ethiopia. Combining all three interventions (diet plus public nutrition plus public health) can avert 20% of stunting in one generation in the Philippines, compared to 28% in Ethiopia. Over four or five generations (maintaining better diet, public nutrition, and public health), girls would grow up less stunted and maternal stature would gradually become less of a limiting factor in birthweight (Pickett et al. 2000). If the same ratios were maintained over time, and if no other unmeasured factors directly influenced stunting, then potentially one could reduce stunting by as much as 45% (9%/20%) through improved diet in the Philippines, and by 50% (14%/28%) in Ethiopia.

### Economic costs of NCDs attributable to unhealthy diets

The largest two components of NCD economic costs are treatment costs and lost productivity due to morbidity and premature mortality, as discussed under Methods. Table 2 presents treatment costs for all NCDs.

**Table 2:** Treatment costs for noncommunicable diseases, Ethiopia 2013 and 2019, and the Philippines 2014 and 2021.

Country/year	Cost of NCD treatment as % GDP	Source	% of NCD treatment costs attributed to 3 metabolic markers^a^	Cost of NCD treatment attributed to 3 metabolic markers as % GDP
Philippines, 2014	1.50%	WHO (2024)	49%	0.74%
Philippines, 2021	2.14%	WHO (2024)	51%	1.09%
Ethiopia, 2013/14^b^	11%×4.08% = 0.45%^c^	Ministry of Health, Ethiopia (2022) and WHO (2024)	33%	0.17%
Ethiopia, 2019/20	24.7%×3.36% = 0.83%^c^	Ministry of Health, Ethiopia (2022) and WHO (2024)	34%	0.29%

^a^Calculated as % of number of NCD deaths attributable to 3 metabolic factors, as a share of all NCD deaths, from Table 3.

^b^Earlier data could not be obtained: costs in 2011 would be lower than in 2013/14 since levels of metabolic markers have been increasing over time.

^c^Calculated using the share of NCDs in health expenditures (first number) from the Ministry of Health, Ethiopia (2022) and share of health expenditure in GDP (second number) from WHO (2024).

As might be expected, the share of NCD treatment costs in GDP is lower in Ethiopia (where metabolic marker levels are lower) than in the Philippines, but in Ethiopia almost doubled between 2013/14 and 2019/20 (Table 2). In the Philippines, the share of NCD treatment costs in GDP also increased between 2014 and 2021 (Table 2), although not as rapidly as in Ethiopia. The Philippines has had a longer experience of increasing metabolic markers than Ethiopia and a similarly longer experience of expanded prevention efforts, such as anti-hypertensives. Not all NCD risks are diet-related. Around half of NCD treatment costs in the Philippines and just less than a third in Ethiopia were associated with conditions for which the three metabolic markers affected by unhealthy diets are risks (Table 2). These shares were increasing slightly over time.

Table 3 provides details of lost productivity (measured using YLLs) due to premature mortality attributable to the three major metabolic markers. These costs are a smaller share of GDP in Ethiopia, which is at an earlier stage in the nutrition transition. The cost share of lost productivity in GDP increased from 4.46% in the Philippines in 2014 to 5.20% of GDP in 2021, while remaining flat in Ethiopia in 2011 (1.41% in 2014% and 1.40% in 2019). Notably, in each country, in each year, losses attributable to high systolic blood pressure account for more losses than high fasting blood glucose and high BMI, combined (Table 3). Supplementary Table S2 presents similar estimates, using DALYs rather than YLLs; however, these numbers are not used here because productivity cost losses due to morbidity and disability are being accounted for instead by estimates of presenteeism and absenteeism.

**Table 3:** Estimates of human and financial costs of premature mortality associated with 3 metabolic markers, Philippines 2014 and 2021 and Ethiopia 2011 and 2019.

Philippines 2014	SBP	FBG	BMI	All 3
Deaths	100 086	49 207	33 694	182 987
YLLs (years of life lost)	2 442 217	1 181 888	1 001 505	4 625 610
Cost USD million of 2014	7001.4	3388.3	2871.2	13 260.9
Cost as % GDP				4.46%

Philippines 2021	SBP	FBG	BMI	All 3
Deaths	123 755	64 717	44 957	233 429
YLLs (years of life lost)	3 004 503	1 555 500	1 319 354	5 879 357
Cost USD million of 2021	10 468.8	5420.0	4597.1	20 485.9
Cost as % GDP				5.20%

Ethiopia 2011	SBP	FBG	BMI	All 3
Deaths	27 913	18 694	8059	54 665
YLLs (years of life lost)	648 511	440 235	228 402	1 317 148
Cost USD million of 2011	222.5	151.0	78.3	451.8
Cost as % GDP				1.41%

Ethiopia 2019	SBP	FBG	BMI	All 3
Deaths	35 250	23 666	10 505	69 421
YLLs (years of life lost)	798 381	531 354	287 302	1 617 038
Cost USD million of 2019	661.6	440.3	238.1	1340.1
Cost as % GDP				1.40%

Notes: SBP refers to high Systolic Blood Pressure; FBG refers to high Fasting Blood Glucose; and BMI refers to high Body Mass Index (overweight and obesity). Number of NCD deaths from all factors were as follows: 364 120 (Philippines 2014); 450 161 (Philippines 2021); 156 675 (Ethiopia 2011); 193 920 (Ethiopia 2019). Derived from IHME VizHub (2021).

Table 4 summarizes the cost results, incorporating the assumption of Okunogbe et al. (2021) regarding additional out-of-pocket costs by patients, presenteeism, and absenteeism, which were not calculated here. As expected, there are differences between Ethiopia and the Philippines. Stunting costs in Ethiopia are a larger proportion of GDP than in the Philippines, but dropping faster. Both treatment and mortality-related costs of NCDs attributable to the three metabolic markers are smaller proportions of GDP in Ethiopia than in the Philippines. In Ethiopia, treatment costs attributable to the three metabolic markers grew rapidly from 2011 to 2019, while the corresponding mortality costs remained flat, as mortality lags rising disease prevalence. The Philippines represents a more mature phase of the evolution of these NCDs, with treatment and mortality costs being higher.

**Table 4:** Summary of costs of stunting and of diet-related NCDs, Philippines and Ethiopia, as % GDP.

Country	Stunting cost as % GDP^a^	Cost of NCDs attributable to 3 metabolic markers, as % GDP			NCD cost allowing for presenteeism, absenteeism, etc.
		Treatment^b^	Mortality^c^	Total (Col 3 + 4)	Col 5*1.27^d^
Philippines	2.83(2013)	0.74 (2013)	4.46 (2013)	5.20	6.60
Philippines	1.9 (2023)	1.09 (2021)	5.20 (2021)	6.29	7.99
Ethiopia	15.54 (2009)	0.17 (2011)	1.41 (2011)	1.58	2.00
Ethiopia	5.9 (2023)	0.29 (2019)	1.40 (2021)	1.69	2.15

^a^Estimates for each component are from the closest year available, from Table 1. Stunting rates do not usually change rapidly year to year, hence costs as % GDP are likely to be slowly declining as rates slowly fall and GDP grows.

^b^From column 5 in Table 2.

^c^From column 5 in Table 3.

^d^As discussed in the text, costs in column 4 are multiplied by 1.27 to allow for costs of absenteeism, presenteeism, and patient out-of-pocket costs, following Okunogbe et al. (2022).

What proportion of the losses attributable to the three metabolic markers can be, in turn, attributed to unhealthy diets? Table 5 summarizes estimates of this proportion for three upper-middle and high-income countries. In the USA and China, prospective cohort studies found multivariable-adjusted hazard ratios or relative risks of around 0.8 for various metabolic risks when comparing either top and bottom GDQS quintiles or ‘high risk’ (GDQS score below 15) versus ‘low risk’ (GDQS score 23 and over) categories of GDQS (defined by Bromage et al. 2021). One study also compared the top and bottom quintiles of GDQS-.

**Table 5:** Summary of the effect of GDQS and/or GDQS- on metabolic markers.

Country, reference	Characteristics of data	Summary of relevant quantitative finding
China, Liu et al. (2024)	Adults, China Health and Nutrition Survey 1997–2015; 3 consecutive days of diet recall; ≤18 years in cohort	A 25% increase in GDQS was associated with a 30% lower risk of new-onset hypertension (RR, 0.70; CI: 0.64–0.76); the multivariable-adjusted relative risk of new onset hypertension was 0.72 (95% CI: 0.62, 0.83) comparing those with high (≥23) versus low (<15) GDQS scores.
USA, Fung et al. (2021)	Adult women, US Nurses Health Survey 1991–2017; type 2 diabetes	Multivariable hazard ratio (HR) for diabetes was 0.83, comparing top and bottom quintiles of GDQS (95% CI: 0.76, 0.91); medians of quintile scores were 29.1 versus 14.5. HR comparing top and bottom quintiles of GDQS- was 0.76 (95% CI: 0.69, 0.84); medians of quintile scores were 11.0 versus 5.5.
China, He et al. (2021)	Adults, China Health and Nutrition Survey round 2010–2012; 3 days of diet recall	Multivariable adjusted odds ratio for metabolic syndrome^a^ was 0.79 comparing top and bottom quintiles of GDQS (95% CI: 0.69, 0.91); means of quintile scores were 29.1 versus 14.5.
Mexico, Angulo et al. (2021)	Adult women, Mexican Teachers’ Cohort, followed for 2 years 2006–2008	Compared to women with little change in GDQS (between −2 and +2), those with the largest increase in GDQS (>5 points) gained less weight and waist circumference; opposite was true for those with the largest decrease in GDQS (> −5 points)

^a^Metabolic syndrome was defined as any three of the following five: high abdominal obesity; high triglycerides; high blood pressure; high fasting blood glucose; and low HDL cholesterol.

Accordingly, the current authors infer that improving diet quality by shifting from high NCD risk diets to low-risk ones, as measured by the GDQS, is potentially associated with a 20% decrease in each of the three metabolic markers. The exact effect on morbidity and mortality depends on how risks are correlated across individuals and their existing levels of risk. In the absence of more detailed data, it is assumed that a 20% decrease in metabolic marker levels is associated with a 20% decrease in treatment costs and productivity losses. Hence, depending on how the level of diet quality (in this case measured by GDQS) is distributed across the population, it is inferred that up to 20% of the costs associated with the three metabolic markers can be attributed to unhealthy diets, while the rest may be attributed to unhealthy lifestyle e.g. limited exercise, use of alcohol and cigarettes, stress, as well as genetic factors. However, no published studies as yet confirm the size of this relationship for a lower-middle-income country (let alone a low-income country such as Ethiopia).

## Discussion

Healthier diets are an important policy goal, and the results suggest that there are important macroeconomic costs of unhealthy diets. The costs of insufficiently diverse diets providing inadequate nutrients remain considerable. However, even low-income countries like Ethiopia are now experiencing rising costs of treatment for NCDs, particularly for cardiovascular disease, suggesting that policy action is urgent, even for countries relatively early in the nutrition transition. While the discussion here has focused on human health, there are obviously wider implications for planetary health (Willett et al. 2019, FAO 2023).

The findings here have implications for methodology, health, and agrifood policy, metrics, and future data collection. With respect to methodology, the cost estimates for diet-related NCDs obtained here with the methodology using three metabolic factors are not greatly different in magnitude compared to those obtained with the methodology using the overweight and obesity methodology (74% for Ethiopia; 120% for the Philippines: Table 6). However, the methodology using three metabolic factors produces estimates only 17% as large for both countries as those obtained with the methodology using 13 individual dietary factors.

**Table 6:** Comparison of estimates of total costs of overweight and obesity and/or diet-related NCD risks among adults from three different methodologies.

Based on overweight and obesity (Okunogbe et al. 2022)	Based on effect of unhealthy diet on 3 diet-related metabolic markers (present study)^a^	Based on GBD 13 GBD dietary risk factors (FAO 2024)
Ethiopia: 0.58% of GDP in 2019	Ethiopia: 0.43% of GDP in 2019	Ethiopia: 2.6% of GDP in 2020
Philippines: 1.34% of GDP in 2019	Philippines: 1.60% of GDP in 2021	Philippines: 9.4% of GDP in 2020
Calculated in USD of 2019	Calculated in USD of 2019 (Ethiopia) and 2021 (Philippines)	Calculated in PPP USD of 2020
Includes treatment costs attributable to overweight and obesity risks, as well as modelled patient travel costs	Includes treatment costs modifiable by diet, attributable to 3 metabolic markers for NCDs	Excludes treatment costs; excludes ‘undernourishment’ costs, provided separately
Methods use losses of productivity due to presenteeism plus absenteeism plus YLL from obesity-attributable diseases due to mortality	Methods sum up 20% of GBD YLL estimates due to 3 metabolic markers, which are independent (no overlap); valued at 1×per capita GDP per YLL; multiplied by 1.27 to include absenteeism, etc.	Methods sum up GBD (2020) DALY estimates due to 13 selected dietary risks (excludes losses due to low fibre and low calcium in diet); an unknown amount of double counting remains
YLL valued at 1 times per capita GDP per YLL	YLL valued at 1 times per capita GDP per YLL	DALY valued using ILO data for annual labour productivity (approx. 1.34 times per capita GDP)

^a^Uses 20% of column 6, Table 4.

With respect to the comparison between the methodologies using overweight and obesity (Okunogbe et al. 2022), arguably, shifting to using the three metabolic factors is preferable. As shown in Fig. 1, using overweight and obesity alone misidentifies the group of those individuals at greatest NCD risk. For example, individuals of Asian origin (Chan et al. 2009) are more prone to diabetes at lower BMI levels than those of Caucasian origin, related to patterns of greater deposition of fat abdominally.

The substantial discrepancy in estimates between models focusing on three metabolic markers and those incorporating multiple (13 or 15) individual dietary risk factors (GBD 2017 Diet Collaborators 2019, FAO 2024) may reflect challenges in appropriately accounting for overlapping effects among correlated dietary exposures. While trial evidence supports robust adjustment when focusing on three metabolic markers, achieving similar control across 13 or 15 dietary factors and health outcomes is less straightforward. For example, GBD 2017 Diet Collaborators (2019) report that 10 dietary factors influence ischaemic heart disease and six affect diabetes, raising questions about how interdependencies are addressed in the modelling framework.

The emphasis on nutrition status versus a more holistic exploration of dietary patterns suggests different policy pathways. Approaches centred on overweight and obesity typically emphasize individual-level behaviours, such as dietary and physical activity patterns, and point towards policies aimed at modifying these behaviours (such as sugar-sweetened beverage taxes, nutritional labelling on packages, and restrictions on advertising to children) and even medical interventions, including weight-loss drugs and bariatric surgery.

In contrast, frameworks that incorporate multiple (13 or 15) individual dietary factors spanning food groups such as whole grains, fruits and vegetables, as well as nutrients and bioactive compounds, including dietary fibre, omega-3 fatty acids, polyunsaturated fatty acids (PUFAs) and transfats are more aligned with research on diet quality and food composition. Our focus on metabolic risk factors and linking these to diet quality measures, such as the GDQS, provides a direct connection to national dietary guidelines. Shifting the lens from nutrition status to dietary intake underscores the explicit role of agrifood policies and the affordability of healthy diets, in conjunction with health sector policies and strategies.

The policy focus is different in the two countries, since they are at different stages in the diet transition (Table 4). In Ethiopia, the most marked shift over the time period examined (in terms of percentage points of cost as a share of GDP) is the decline in economic costs of stunting, whereas in the Philippines, it is the increase in economic costs of NCDs attributable to diet. The country context section noted that, whereas 48.1% of the population of the Philippines could not afford a ‘healthy’ diet, this figure was 83.9% in Ethiopia. In Ethiopia, increasing dietary diversity by adding healthy food groups is important, whereas the Philippines faces a more complex task, namely switching consumption away from unhealthy food groups such as refined grains and sweets towards healthier ones like whole grains, fruits, and vegetables.

In the Philippines, preliminary results from the 2018, 2019, and 2021 National Nutrition Surveys (Authors 2025), using the subsample of individuals who provided 24-hour dietary recall for two days (136 338 individuals aged 18 and over), suggest that the mean GDQS is 15.8 (95% CI 15.7–16.0). This is barely above 15, and individuals with GDQS scores below 15 are judged to be at ‘high’ NCD risk. The low score is due to limited consumption of healthy foods (GDQS + is 5.0) rather than to unusually high consumption of unhealthy foods (GDQS- is 10.8).

Intake (2025) shows that the Philippines in 2001 exhibited lower scores than Ethiopia in 2013 on the overall diet quality metric (GDQS) as well as both submetrics (GDQS+ and GDQS-, denoting healthy and unhealthy food groups). This has implications in both countries for increased production of fruits and vegetables, for support for production of whole or less-highly-processed grains, for improvements in storage and distribution such that healthy perishable foods are available and affordable, and for using health policy to promote consumption of these foods.

There are implications for the use of metrics. The analysis here relied on evidence for upper-middle-income and high-income countries to estimate the proportion of NCD risks attributable to diet quality (as measured by the GDQS). The Philippines is one of the only low and lower-middle-income countries where the national food consumption survey also contains information on each of BMI, blood glucose, and hypertension, such that the same proportion could potentially be estimated, a subject of ongoing work (Authors 2025). It remains to be seen whether the relationship between current diet quality and metabolic measures is sufficiently robust to be estimated using two-day diet recall, or whether either three-day diet recall data is necessary, as in China (He et al. 2021, Liu et al. 2024), or cohort data as in Mexico (Angulo et al. 2021) and the USA (Fung et al. 2021).

Additional work on diet metrics would also be beneficial. A recent study (Hanley-Cook et al. 2024) suggests that composite metrics based on both healthy and unhealthy food groups, such as the GDQS and the GDR score, have less discriminatory capability across countries of different levels of income, and recommends further work using the submetrics for unhealthy foods (e.g. GDQS-). This is consistent with Fung *et al.*’s (2021) findings for the US, that the analysis using quintiles of the GDQS was associated with a stronger effect on the onset of type 2 diabetes than when using the composite indicator GDQS.

Strengths of our approach include that the calculations involve a more complete modelling framework that traces the pathway from dietary patterns to three key metabolic markers, and ultimately to disease outcomes. This contrasts with a ‘black box’ approach that aggregates multiple markers without explicitly accounting for overlaps and interdependencies among dietary risk factors. Limitations of our approach include that although the GBD 2021 Risk Factor Collaborators (2024) provides estimated ranges for the deaths and YLLs, there are no easily-calculated ranges for the other assumptions (the share of treatment costs attributed to diet-sensitive metabolic markers, the sensitivity of metabolic markers to changes in the measure of diet quality, and the value of presenteeism, absenteeism and out-of-pocket costs). Hence, no uncertainty estimate is calculated, and the resulting estimates are orders of magnitude only. Another limitation is that data for stunting are from similar but not necessarily identical years as data for metabolic markers.

Obviously, healthier diets are only one component of healthier lifestyles. Estimates here suggest that about half of the costs of stunting are diet-related, while public nutrition and public health policies combined are of roughly equal importance to diet. Table 4 suggests that up to 20% of the costs of those NCDs sensitive to diet could be alleviated by healthy diets (potentially more if salt intake, which is not readily measured in dietary recall studies, could also be reduced). Other factors such as alcohol intake, smoking, sedentary lifestyle, and stress are also important.

Unhealthy diets impose large and growing costs to the global economy. Reining in the human and economic costs will require co-ordinated policy effort from the health, nutrition, and agrifood sectors, and this needs to begin now in countries at all income levels.

## Supplementary Material

czag018_Supplementary_Data

## Data Availability

Data used are publicly available from the sources cited.

## Reflexivity statement

Four of the nine authors are nationals of the Philippines; six are women; three are early-career scholars (recent PhDs) and two are junior scholars. Another author has periodically worked in and on the Philippines throughout several decades. All of the authors contributed to capacity-building workshops for colleagues at the Department of Science and Technology Food and Nutrition Research Institute (Philippines) in the course of undertaking the research, and seven of the nine authors participated in two week-long in-person policy consultations in the Philippines. One author has worked extensively in Ethiopia, participated in two week-long in-person policy consultations in Ethiopia in the course of undertaking the research (joined by another two authors for the second consultation) and is co-authoring a separate study on changing diet patterns between 2011 and 2021/2 with Ethiopian colleagues.
