# Supplementary material for: How much can healthier diets reduce future economic and human costs? Results from Ethiopia and the Philippines
Source: Health Policy Plan. 2026 Feb 10;41(4):599–611. doi: 10.1093/heapol/czag018 (PMC13089433; doi:10.1093/heapol/czag018)
Supplement: czag018_Supplementary_Data [file czag018_supplementary_data.zip › SuppTable1HPPDbleSpace.docx]

**Supplementary table 1. Baseline scenarios for coverage of interventions for Lives Saved Tool (LiST) modelling of stunting outcomes.**

| Interventions | | Assumed 2024 coverage | |
| --- | --- | --- | --- |
|  |  | **Ethiopia**  **(%)** | **Philippines**  **(%)** |
| Diet-related | Folic acid fortification among WRA | 0 | 0 |
|  | Iron fortification among WRA | 0 | 35.0 |
|  | Balanced energy-protein supp in pregnancy | 0 | 0 |
|  | Exclusive breastfeeding (0-5 months) | 73.1 (<1m)  56.0 (1-5m) | 54.4 (<1m)  38.3 (1-5m) |
|  | Appropriate complementary feeding (6-23 months) | 11.3 | 37.3 |
| Public Nutrition | Iron-Folic Acid or Multiple Micronutrition Supplementation in pregnancy | 10.6 | 56.1 |
|  | Calcium in pregnancy | 0 | 0 |
|  | 6-59 months Vit A supp | 73 | 29 |
|  | 12-59 months zinc supp | 0 | 0 |
| Public Health | Syphilis detection and treatment | 41.1 | 43.5 |
|  | Progesterone for at-risk births | 0 | 0 |
|  | Low-dose aspirin | 0 | 0 |
|  | Improved sanitation | 9.3 | 84.8 |
|  | Improved water (piped + filtered) | 44.1 piped  1.2 filtered | 66.5 piped  2.5 filtered |
|  | Handwashing with soap | 8.3 | 81.8 |
|  | Rotavirus vaccine | 65 | 0 |
| ^1^ Based on most recent data sources available in the online version of the LiST modelling tool (<https://list.spectrumweb.org>); primarily DHS 2019 for Ethiopia and DHS 2022 for the Philippines | | | |
