# Supplementary material for: How much can healthier diets reduce future economic and human costs? Results from Ethiopia and the Philippines
Source: Health Policy Plan. 2026 Feb 10;41(4):599–611. doi: 10.1093/heapol/czag018 (PMC13089433; doi:10.1093/heapol/czag018)
Supplement: czag018_Supplementary_Data [file czag018_supplementary_data.zip › SuppTable2HPP.docx]

**Supplementary Table 2. Estimates of human and financial costs of disability and premature mortality associated with 3 metabolic markers, Philippines 2014 and 2021 and Ethiopia 2011 and 2019 using DALYs.**

| Philippines 2014 | SBP | FBG | BMI | All 3 |
| --- | --- | --- | --- | --- |
| Deaths | 100,086 | 49,207 | 33,694 | 182,987 |
| DALYs | 2,553,307 | 1,482,666 | 1,221,366 | 5,257,339 |
| Cost USD million of 2014 | 7,319.9 | 4,250.6 | 3,501.5 | 15,071.9 |
| Cost as % GDP |  |  |  | **5.07%** |
| Philippines 2021 | **SBP** | **FBG** | **BMI** | **All 3** |
| Deaths | 123,755 | 64,717 | 44,957 | 233,429 |
| DALYs | 3,144,496 | 1,981,055 | 1,632,738 | 6,758,289 |
| Cost USD million of 2021 | 10,956.6 | 6,902.8 | 5,689.1 | 23,548.5 |
| Cost as % GDP |  |  |  | **5.98%** |
| Ethiopia 2011 | **SBP** | **FBG** | **BMI** | **All 3** |
| Deaths | 27,913 | 18,694 | 8,059 | 54,665 |
| DALYs | 682,457 | 565,344 | 292,816 | 1,540,618 |
| Cost USD million of 2011 | 234.1 | 193.9 | 100.4 | 528.5 |
| Cost as % GDP |  |  |  | **1.65%** |
| Ethiopia 2019 | **SBP** | **FBG** | **BMI** | **All 3** |
| Deaths | 35,250 | 23,666 | 10,505 | 69,421 |
| DALYs | 846,027 | 712,027 | 382,452 | 1,940,506 |
| Cost USD million of 2019 | 701.1 | 590.1 | 316.9 | 1,608.1 |
| Cost as % GDP |  |  |  | **1.68%** |

Notes: SBP refers to high Systolic Blood Pressure; FBG refers to high Fasting Blood Glucose; and BMI refers to high Body Mass Index (overweight and obesity). DALYs refers to Disability-Adjusted Life Years.
